# A savings intervention to reduce men’s engagement in HIV risk behaviors: study protocol for a randomized controlled trial

**DOI:** 10.1186/s13063-022-06927-0

**Published:** 2022-12-16

**Authors:** Teniola I. Egbe, Ouma Dan Omollo, Julius Oduor Wesonga, Elizabeth F. Bair, Averi Chakrabarti, Mary E. Putt, Connie L. Celum, Carol S. Camlin, Sue Napierala, Kawango Agot, Harsha Thirumurthy

**Affiliations:** 1 Department of Medical Ethics and Health Policy, Perelman School of Medicine, University of Pennsylvania, Philadelphia, PA USA; 2 Impact Research and Development Organization, Kisumu, Kenya; 3 Department of Biostatistics, Epidemiology & Informatics, Perelman School of Medicine, Philadelphia, PA USA; 4 Department of Global Health, University of Washington, Seattle, WA USA; 5 Department of Obstetrics, Gynecology and Reproductive Sciences, University of California San Francisco, San Francisco, CA USA; 6 Women’s Global Health Imperative, RTI, Berkeley, CA USA

**Keywords:** HIV/STI prevention, Transactional sex, Economic interventions, Behavioral economics, Savings, Kenya, Men, Randomized controlled trial

## Abstract

**Background:**

In much of eastern and southern Africa, the incidence of HIV and other sexually transmitted infections (STIs) remains high despite the scale-up of promising biomedical and behavioral interventions. Studies have documented the crucial role of transactional sex—the exchange of money, material support, or goods, in sexual relationships—and heavy alcohol use in contributing to men’s and women’s health outcomes. Existing policy responses to this challenge have largely focused on women, through the provision of pre-exposure prophylaxis (PrEP) or structural interventions such as education subsidies and cash transfers. However, the effectiveness of these interventions has been hindered by the relative lack of interventions and programs targeting men’s behavior. We describe the protocol for a study that will test an economic intervention designed to reduce men’s engagement in HIV/STI-related risk behaviors in Kenya.

**Methods:**

We will conduct a randomized controlled trial among income-earning men in Kenya who are aged 18–39 years and self-report alcohol use and engagement in transactional sex. The study will enroll 1500 participants and randomize them to a control group or savings group. The savings group will receive access to a savings account that includes lottery-based incentives to save money regularly, opportunities to develop savings goals/strategies, and text message reminders about their savings goals. The control group will receive basic health education. Over a period of 24 months, we will collect qualitative and quantitative data from participants and a subset of their female partners. Participants will also be tested for HIV and other STIs at baseline, 12, and 24 months.

**Discussion:**

The findings from this study have the potential to address a missing element of HIV/STI prevention efforts in sub-Saharan Africa by promoting upstream and forward-looking behavior and reducing the risk of acquiring HIV/STIs in a high HIV/STI burden setting. If this study is effective, it is an innovative approach that could be scaled up and could have great potential for scientific and public health impact in Kenya.

**Trial registration:**

ClinicalTrials.govNCT05385484. Registered on May 23, 2022

## Administrative information

Note: the numbers in curly brackets in this protocol refer to SPIRIT checklist item numbers. The order of the items has been modified to group similar items (see http://www.equator-network.org/reporting-guidelines/spirit-2013-statement-defining-standard-protocol-items-for-clinical-trials/).

**Table:** 

Title	A savings intervention to reduce men’s engagement in HIV risk behaviors: study protocol for a randomized controlled trial
Trial registration {2a and 2b}.	Study was registered on ClinicalTrials.gov on May 23,2022. Link: https://clinicaltrials.gov/ct2/show/NCT05385484. Trial registration number: NCT05385484
Protocol version	Version 4.0 on September 15, 2022
Funding {4}	*Eunice Kennedy Shriver* National Institute of Child Health and Human Development
Author details {5a}	Teniola I. Egbe, MPH MBE^1^, Ouma Dan Omollo, MPH^2^, Julius Oduor Wesonga, MScE^2^, Elizabeth F. Bair, MS^1^, Averi Chakrabarti, PhD^1^, Mary E. Putt, PhD, ScD^3^; Connie L. Celum, MD^4^, Carol Camlin, PhD^5^, Sue Napierala, PhD^6^, Kawango Agot PhD, MPH^2^, and Harsha Thirumurthy, PhD^1^ ^1^ Department of Medical Ethics and Health Policy, Perelman School of Medicine, University of Pennsylvania, Philadelphia, PA, United States of America; ^2^ Impact Research and Development Organization, Kisumu, Kenya; ^3^ Department of Biostatistics, Epidemiology & Informatics, Perelman School of Medicine, Philadelphia, PA, United States of America; ^4^ Department of Global Health, University of Washington, Seattle, WA, United States of America; ^5^ Department of Obstetrics, Gynecology and Reproductive Sciences, University of California San Francisco, San Francisco, CA, United States of America; ^6^ Women's Global Health Imperative, RTI International, Berkeley, CA, United States of America
Name and contact information for the trial sponsor {5b}	Eunice Kennedy Shriver National Institute of Child Health and Human Development, P.O. Box 3006, Rockville, MD 20847
Role of sponsor {5c}	The sponsor had no role in the study design, collection, management, analysis, and interpretation of data; writing of the report; and the decision to submit the report for publication.

## Introduction

### Background and rationale {6a}

HIV incidence in sub-Saharan Africa (SSA) remains above elimination levels despite the scale-up of evidence-based, biomedical interventions like antiretroviral therapy (ART), medical male circumcision, and pre-exposure prophylaxis (PrEP) [1]. While these interventions are essential, there is a parallel need for behavioral interventions that address upstream drivers of HIV risk like transactional sex and heavy alcohol use [2, 3, 4]. Age-disparate relationships, including those involving transactional sex—the exchange of money, material support, or goods for sex—are considered to be a leading reason why HIV incidence remains exceptionally high, particularly among adolescent girls and young women. Many studies have reported a high prevalence of transactional sex among both men and women in SSA [2, 5, 6]. In Kenya’s Nyanza region, one study found 65% of men reporting engagement in transactional sex [5]. In a recent pilot study done in Kenya, 47% of men reported paying for sex in the past 3 months [7].

Efforts to reduce transactional sex and HIV/STI risk in SSA have largely focused on women, with the goal of either reducing risks associated with their engagement in transactional sex (e.g., through the provision of health services like PrEP or STI treatment or promotion of condom use) or reducing their reliance on transactional sex altogether (e.g., through cash transfers). For example, UNAIDS recommendations include PrEP, education subsidies, and cash transfers for economic empowerment [4, 8]. However, the effectiveness of these interventions has been hindered by the relative lack of programs that target men’s sexual behaviors.

Heavy alcohol consumption is another important risk factor for HIV that has not been addressed with scalable interventions. Men in SSA have a 21–60% prevalence of heavy alcohol use (based on varying definitions), with the highest prevalence among those aged 18–39 years [9, 10]. Heavy alcohol use is associated with 50–70% higher HIV risk—because of the disinhibiting effect of alcohol on sexual behavior and because alcohol outlets draw individuals with risk-seeking preferences [3, 11]. These data underscore the need for HIV/STI prevention interventions that also address heavy alcohol use.

This study will test an economic intervention that has the potential to reduce men’s engagement in transactional sex and other HIV risk behaviors such as alcohol use. Recognizing that men who engage in transactional sex and alcohol use have disposable income but lack savings opportunities or incentives to save, the study will test the effectiveness of a savings intervention that includes incentives and reminders to save. The intervention is motivated by empirical evidence that men are more risk-seeking [12, 13] and less future-oriented [14] than women. It is also inspired by emerging evidence on how access to saving products can increase savings and investment and build future orientation (i.e., reduce future discounting) [15].

As mobile banking services become widely available in SSA, savings interventions have significant untapped potential to motivate men to reduce their spending on risky behaviors and save more of their income for the future. Savings interventions can influence men’s behavior through a direct economic mechanism (incentives to decrease expenditures on transactional sex and alcohol while increasing savings for the future) and a psychological mechanism (increasing future orientation). Conceived in this way, savings interventions have a strong theoretical premise and are ideally suited to reducing income-earning men’s engagement in HIV/STI risk behaviors. They can complement biomedical and behavioral approaches that focus on women (e.g., PrEP, conditional cash transfers, etc.) in order to reduce the risks associated with transactional sex.

Building on a promising pilot randomized trial among men in Kenya, this large-scale randomized controlled trial will determine the effect of a savings intervention on the composite incidence of HIV and other STIs as well as on health and economic behavior among men engaged in transactional sex and heavy alcohol use. The study will also use mixed qualitative and quantitative methods to study mechanisms through which the intervention affects men’s health and economic outcomes.

### Objectives {7}

The primary objective of the *Akiba* study (Kiswahili for savings) is to determine the effect of a savings intervention on the composite incidence of HIV/STIs over 24 months. The secondary objective will be to quantitatively and qualitatively assess mechanisms of behavior change among participants and a sample of their female partners.

### Trial design {16}

The *Akiba* study is a single-center two-arm parallel group trial designed to test the superiority of a behavioral intervention over control. Participants will be individually randomized 1:1 to either control, comprised of basic health education, or an intervention involving the establishment of a savings account, lottery-based incentives to save, and behavioral nudges to promote savings through goal setting and reminders.

## Methods: participants, interventions, and outcomes

### Study setting {9}

This study will take place in Siaya County, Kenya, which has a population of about 1 million. HIV prevalence in Siaya County is the third highest in Kenya, at 15.3%, and STI incidence is also high [16, 17, 18]. Although there is high poverty in the region, many men earn a steady income through fishing, transport sector work, and informal labor. Studies have also documented a high prevalence of heavy alcohol use and transactional sex [19].

### Eligibility criteria {10}

Men will be screened for several eligibility criteria: age 18–39 years, self-reported alcohol or other substance use in the past month, self-reported engagement in transactional sex in the past 3 months, earning regular income, and ownership of a mobile phone. Men must also have or be willing to create a Kenya Revenue Authority PIN code and have a national identification card, which are required to open a bank account. They must also be willing to open an account (or currently have an account) at the bank that will be partnering with the study.

HIV/STI status will not be an eligibility criterion. Men who are seropositive for HIV at baseline will be eligible if they meet all other criteria as the intervention is intended to be HIV status-neutral. Including these men is important for the generalizability of findings. For the qualitative portion of this study, we will recruit adult female partners of a subset of male study participants.

### Who will take informed consent? {26a}

Informed consent will be obtained by trained research assistants from all study participants.

### Additional consent provisions for collection and use of participant data and biological specimens {26b}

Verbal informed consent will be obtained prior to written informed consent in order to screen men for eligibility for the study. Men who meet eligibility criteria will be asked to provide informed consent before they are enrolled in the study. The consent process will be conducted in English, Kiswahili, or Dholuo (as per men’s preferences).

Men and women invited to take part in the qualitative sub-study will also be given a detailed description about the qualitative study procedures, potential risks and benefits, and compensation for participation. Research assistants will emphasize that data collected from all participants (male and female) will be strictly confidential and will not be shared with their partner. A separate informed consent process for the in-depth interviews (IDIs) will be administered when these interviews are conducted.

## Interventions

Participants will be randomized in a 1:1 ratio to a control or intervention group. Participants will be followed for a period of approximately 24 months, with six-monthly study visits at 6, 12, 18, and 24 months. Participants will be tested for HIV and other STIs at baseline, 12, and 24 months. Testing will not be repeated for those who test positive for HIV or for an untreatable STI at subsequent visits. We will conduct longitudinal IDIs at 12 and 24 months, in a sub-sample of men and their primary female partners. These IDIs will provide a nuanced understanding of changes in men’s behavior and their future orientation and female partners’ perceptions about changes in men’s behavior. The emphasis will be to collect detailed narratives from participants in order to understand mechanisms through which the intervention influences health and economic outcomes and to assess potential “spillover effects” of the intervention (benefits or harms) across multiple domains and populations. IDIs with women will also assess feasibility and interest in the savings intervention, which can inform a future study that examines whether the effects on HIV/STI incidence are larger when the intervention is offered to both men and women.

### Explanation for the choice of comparators {6b}

Participants in the control group will receive basic health education as well as basic information on the importance of saving for the future.

### Intervention description {11a}

Participants in the intervention group will receive assistance from study staff and local bank staff in Kenya in opening and using a savings account. In addition, they will receive an education session that emphasizes the importance of saving for the future, provides a tutorial on how to earn interest on savings, and addresses common barriers to saving (lack of trust, lack of information, etc.). Participants’ bank accounts will be endowed with an initial deposit of 500 Kenyan Shillings (≈4USD). At the time of enrollment, research assistants will discuss with participants their reasons for saving, their savings goals, and the challenges to saving money each month. The research assistants will encourage participants to develop a savings goal amount and to define a purpose(s) for saving (e.g., future investment in farming or businesses, household expenses on durable goods, children’s education, etc.) and concrete strategies to save, including developing a timeframe and amount needed to save to achieve savings/investment goals. This activity is based on prior research demonstrating that setting savings goals and implementation plans can increase the motivation to save [20, 21].

Participants will have a chance of winning monetary prizes each month based on the amount by which their account balance goes up during the month. Each month’s lottery will have several prizes, including a grand prize and several medium and small prizes ranging from about 500 to 10,000 Shillings. These prize amounts were determined after pilot testing of men’s preferences for different amounts of lottery prizes and probabilities. Prizes will be deposited directly into bank accounts and winning participants will be informed by text message. Participants who do not win a prize in a given month may receive text messages encouraging them to save and reminding them of the goal they had previously set. In addition, participants may receive text messages each month reminding them of savings goals, strategies, and the lottery-based incentives for saving. This approach builds on promising findings in the savings literature which suggest that reminders might help promote savings [21].

### Criteria for discontinuing or modifying allocated interventions {11b}

Participants will be informed that they may withdraw from the study at any time. No additional data will be collected from participants who withdraw from the study.

A participant may also be discontinued from the study at the discretion of the principal investigator for lack of adherence to intervention or study procedures or visit schedules or due to adverse events. The principal investigator or the sponsor may also withdraw participants who violate the study plan, to protect the participant for reasons related to safety or for administrative reasons.

### Strategies to improve adherence to interventions {11c}

To improve adherence to the intervention, bank balances will be collected and reviewed on a monthly basis from the local bank where participants opened their accounts. If balances drop below 500 KSH (initial deposit) provided to participants by the study, the research assistants will contact the participants to inquire about the reason for this decrease and encourage them to save. Furthermore, staff will contact participants before their visits to remind them of their next visit and provide them an opportunity to meet in a more convenient location. All efforts will be made to promptly reschedule appointments with participants who have missed a visit (within a month of the initial appointment) so as to ensure high adherence to the intervention. In addition, to encourage participants to save towards their goals, they may receive text message nudges throughout the study.

### Relevant concomitant care permitted or prohibited during the trial {11d}

There is no relevant concomitant care permitted or prohibited during the trial.

### Provisions for post-trial care {30}

We do not anticipate any harm from trial participation. There are no plans for any post-trial care.

### Outcomes {12}

The primary outcome will be HIV/STI incidence over 24 months, defined as the composite incidence of at least one of HIV or other STIs (herpes simplex virus type 2 (HSV-2), *Neisseria gonorrhoeae* (NG), and *Chlamydia trachomatis* (CT)). The combination of all STIs (including HIV) into a composite variable is justified as the risk factors for these STIs are similar. Compared to a strategy of considering the individual infections as separate variables, using the composite variable (any incident HIV/STI infection) will increase the number of events and hence our ability to detect an effect of the intervention. For participants who are HIV-positive at baseline (~20%) or HSV-2-positive (~40%), the outcome will be defined over all STIs other than HIV and/or HSV-2.

Secondary economic outcomes include total savings and investment at 24 months, total formal savings (i.e., the sum of savings balances in all formal bank accounts), total household assets, human capital investments, housing improvements, expenditures on transactional sex and alcohol, and expenditures on gambling. Secondary health outcomes include measures of sexual behavior—number of sexual partners in the past 3 months, condom use at last sex, engagement in transactional sex in the past 3 months, number of transactional partners in past 3 months—alcohol use measured by the Alcohol Use Disorders Identification Test-Concise (AUDIT-C) scale, incidence of STIs (including HIV and excluding HSV-2) between 0–12 months and 12–24 months, and self-reported engagement in care among participants who are HIV-positive. We will also investigate mediators such as future orientation and financial security.

### Participant timeline {13}

Participants enrolled in the study will be administered a questionnaire at baseline, 6, 12,18, and 24 months. All questionnaires will be completed within a window of 1 month before or after each interval. We will evaluate the presence of HIV, NG, and CT at baseline, 12 months, and 24 months. We will also test for HSV-2 at baseline and 24 months. We will conduct longitudinal IDIs at 12 and 24 months in a sub-sample of men and their primary female partners. The project schedule of enrolment, intervention, and assessments are detailed in Table 1.

**Table 1:**
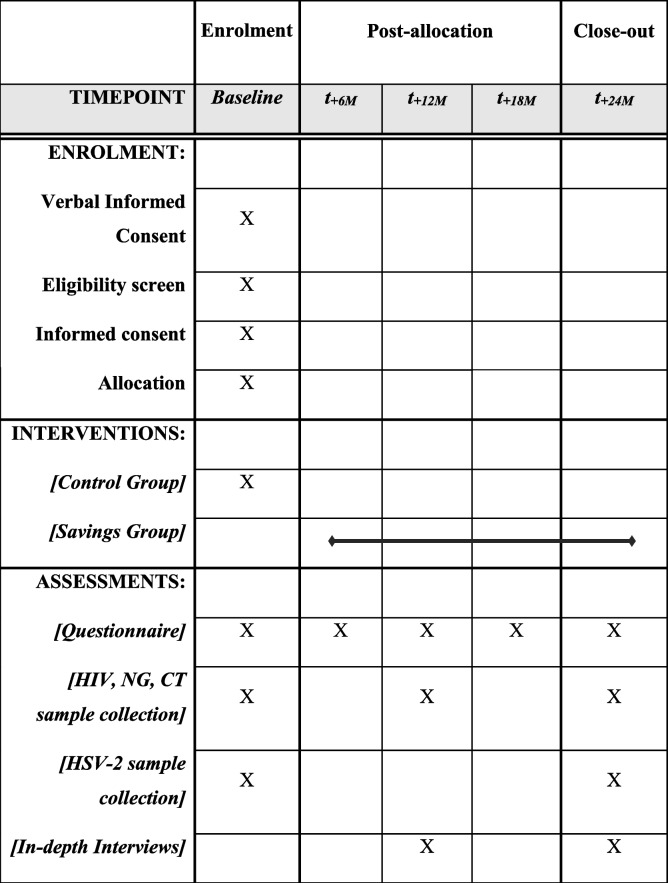
SPIRIT flow diagram of the schedule of enrolment, interventions, and assessments in the Akiba study

### Sample size {14}

Power calculations assumed that composite HIV/STI incidence will be 15–20% per year, with contributions from each STI as follows. HSV-2: data from Kenyan men show annual HSV-2 incidence among all men (adjusting for ~40% HSV-2 baseline prevalence) of 6–10% [22]. HSV-2 incidence has been even higher among young men and higher-risk men (e.g., recent data for 18–35-year-old men from the Afya Jozi Afya Jamii study). CT/NG: composite CT/NG annual incidence was 7–8% in studies among Kenyan men [23], though some studies have reported higher incidence [24]. HIV: annual incidence of <1% is assumed from recent studies, and our anticipated incidence adjusts for baseline prevalence [18]. Accounting for co-infection with multiple STIs and baseline prevalence of HIV and HSV-2 (20% and 40%), our anticipated composite HIV/STI incidence is conservative.

To have adequate statistical power to detect a meaningful reduction in composite HIV/STI incidence over 24 months, we will enroll a total of 1500 participants (750 per study arm). Table 2 shows that under difference assumptions of annual composite incidence in the control group, the study will have 80% power to detect a relative reduction of 21–25%. We included a conservative assumption of 20% attrition over 24 months (expected to be lower given our prior studies). Calculations are based on a two-group chi-squared test of proportions.

**Table 2:** Detectable reduction in composite HIV/STI incidence over 24 months (*N*=1500, 80% power, 20% attrition, two-sided *α*=.05)

	Scenario		
Composite HIV/STI incidence rate in the control group (per 12 months)	15%	17%	20%
Composite HIV/STI incidence rate in the control group (per 24 months)	28%	31%	36%
Detectable relative reduction in composite incidence (relative to control)^a^	0.25	0.23	0.21

^a^1 − (rate in the intervention group)/(rate in the control group)

For the qualitative portion of this study, we will use structured and theoretical sampling approaches to compose a cohort of ~25 men from the overall study population. First, a structured sample of around *n*=15 participants will be randomly selected from the study sample, with over-sampling from the intervention arm given that the main objective of this study component is to understand how the intervention influences behavior. Control arm participants will be included to gain further insight on socio-ecological factors as well as health and economic decision-making. Second, using 12-month outcome data, we will systematically select around 10 additional men from the intervention arm to ensure the sample includes men who have high savings (>median) and low or no savings (<median), thus capturing diverse experiences. We will also seek to have representation of men who report high and low levels of sexual risk behavior at 12 months. Finally, we will employ additional theoretical sampling as needed on the basis of emergent qualitative data at 12 months to ensure theoretical saturation of the data.

### Recruitment {15}

Participants will be recruited from a small number of communities within Bondo sub-County of Siaya County. Study staff members will meet with local leaders and provide general information on the study. Subsequently, the study team will map out all stores, stages, or workshops within the communities which men frequent, work in, or own. This is ideally suited for recruiting high-risk men who earn steady cash income. Study staff will provide general information on the study to men and refer them to nearby study offices where research assistants will screen them for eligibility.

## Assignment of interventions: allocation

### Sequence generation {16a}

Once a participant has completed their baseline questionnaire and sample collection, they will be stratified on baseline HIV status and within each stratum, they will be randomized (1:1) to an intervention or control arm using a computer-generated sequence with blocks of sizes of 10.

### Concealment mechanism {16b}

Treatment allocation will be revealed to participants and research assistants after they obtain the results of their HIV test by opening a sealed envelope that corresponds with the results of their HIV status (one set of envelopes for those that test negative and one for those that test positive/indeterminate). Each envelope will contain a sheet of paper that states whether a participant is in the control group or intervention group.

### Implementation {16c}

The allocation sequence was generated by the principal investigator. Participants are randomized to a trial arm based on the results of their HIV baseline test results.

## Assignment of interventions: blinding

### Who will be blinded {17a}

Given the nature of this study, blinding is not possible for study staff nor participants, but the principal investigator and co-investigators will remain blinded during the course of this study.

### Procedure for unblinding if needed {17b}

N/A: Once the participants open the envelope, the study team and participants will know which trial arm they belong to. In the event of an adverse event or social harm, the principal investigator and other co-investigators may be unblinded.

## Data collection and management

### Plans for assessment and collection of outcomes {18a}

#### Data collection, management, and safety

At baseline and each 6-monthly follow-up visit, research assistants will administer a questionnaire to obtain information on participants’ employment, income, assets, formal and informal savings, expenditures on food items and non-food items (including alcohol, transactional sex, gambling), self-reported sexual behavior, alcohol use, and HIV and STI risk perceptions. The questionnaire will also measure various decision-making characteristics (e.g., future orientation) that are relevant study mediators.

Well-being will be measured by the Cantril Scale [25], alcohol use will be measured by the Audit-C scale [26] which is scored on a scale of 0–12 (higher values represent worse outcomes), food insecurity will be measured by the Household Food Insecurity Access Scale [27], financial security will be measured by the Financial Security Scale [28], and stress level will be measured by Cohen’s Perceived Stress Scale [29]. In addition to using the validated scales above, some questions in our questionnaire were adapted from the World Bank’s Living Standards Measurement Survey [30], the Transfer Project’s surveys [31], and the Kenya Demographic and Health Surveys [32].

Within a month’s period, all study staff will be trained and complete all necessary certificates in accordance with Kenya’s Ministry of Health. All HIV/STI testing will be performed by trained research assistants certified by Kenya’s National AIDS and STI Control Programme (NASCOP). HIV testing will be performed in accordance with HIV antibody testing algorithms recommended by Kenya’s Ministry of Health [33]. HSV-2 samples will be tested for HSV-2 antibodies using Kalon HSV-2 IgG ELISA, which has higher sensitivity than other HSV-2 enzyme immunoassays [34, 35] and according to manufacturer instructions and recommended cut-off for HSV-2 positivity. CT/NG testing will be performed with the Cepheid GeneXpert rapid PCR test for the detection of CT and NG.

### Plans to promote participant retention and complete follow-up {18b}

Extensive efforts will be put in place to ensure that enrolled participants are retained in the study. Research assistants will complete a locator form from each participant that includes any phone numbers that could be used to contact participants as well as detailed directions to their home and workplace. Additionally, we will arrange to meet participants at alternate locations if they have moved away from their initial residence or are temporarily away. We will also contact participants at 3, 9, 15, and 21 months for a brief survey, which will be useful for promoting retention in the study. Similar to past studies involving long-term follow-up, we seek to achieve retention rates of 85–95% per year using such a combination of retention strategies.

### Data management {19}

We have developed standard operating procedures for data security and confidentiality procedures at collection, transfer, entry, and storage levels and will train all staff members who have access to confidential study data. Study tablets used for data collection will be password protected. Data will be collected using Research Electronic Data Capture (REDCap) which is a secure online tool for data collection and storage. To ensure the integrity of the data, regular quality checks will be performed to assess for missing data and invalid field entries. Completed surveys will be stored as encrypted data on the password-protected REDCap application. All electronic study data will be stored on a secure terminal server at the University of Pennsylvania with access limited only to authorized staff. All computers and servers will be encrypted and password-protected with limited access. All electronic information will be recorded using study identification numbers, rather than participant names.

Physical data collection forms, such as the informed consent form and locator form will be stored at Impact Research & Development Organization (IRDO) headquarters in Kisumu as they are implementing partner. All sensitive information will be stored separately from any identifying information to prevent breach of confidentiality. After the end of the study, electronic data will be kept for up to 5 years on the University of Pennsylvania terminal server and up to 2 years for paper forms, including consent forms, at the IRDO facility in Kenya.

### Confidentiality {27}

Unless specifically requested by a participant, all study visits will be conducted at designated study offices. This will allow for increased privacy. All study offices will have private rooms for screening and enrollment. Conducting visits at study offices also reduces the concern that participants’ household members may learn about their alcohol use or engagement in transactional sex. All study staff have been trained about confidentiality.

Rapid HIV testing and sample collection for STI testing will take place at study offices. Positive STI test results will be shared with participants and those testing positive for curable STIs will be asked to come to an IRDO study site for treatment. The reason for the visit will be unknown to non-study members, and the visit will be conducted in a confidential manner. Test results will be linked to participants only by their unique study identification number and not by name. At all times possible, the samples and test results will be kept in a secure location and only accessible by authorized research staff. Authorized research staff with the International Air Transport Association (IATA, transportation of dangerous goods/training in biomedical safety procedures) will conduct the transfer of urine and blood samples from the field site to the IRDO laboratory in Kisumu.

Aggregate statistics on HIV tests will be reported to the government health facility serving the catchment area where testing is done, as per Kenyan guidelines; participants’ identity will not be disclosed. STI tests are not reportable to local government facilities since we will not be using Kenyan Ministry of Health test kits.

### Plans for collection, laboratory evaluation, and storage of biological specimens for genetic or molecular analysis in this trial/future use {33}

Samples collected will be stored, transported, and tested in accordance with local guidelines.

HIV testing will be completed using rapid tests (determine and first response) in accordance with NASCOP’s new HIV testing algorithm. For HSV-2 testing, a minimum of 6 ml of whole blood will be collected in SSTs or red top vacutainer tubes. For CT/NG testing, participants will be asked to provide 10–20 ml of first void or “first catch” urine into a screw-capped urine container free of preservatives. Participants will be advised not to cleanse the tip of the penis area prior to collecting the urine sample. All samples collected will be labeled with participant details including date/time of collection and the collector’s initials or signature. Samples will be placed on a rack inside a cooler box and transported at ambient temperature. The shipment temperature will be monitored from the field to the laboratory by use of thermometers or data loggers placed inside the cooler box.

Participant’s information and samples will not be stored or shared for future research purposes.

## Statistical methods

### Statistical methods for primary and secondary outcomes {20a}

For the primary outcome, we will estimate the odds ratio for treatment versus control arms of at least one STI/HIV infection at 24 months, using a logistic regression model and controlling for baseline HIV and HSV-2 status. A two-sided type I error rate of 0.05 will be used for hypothesis testing of the treatment effect.

The secondary analyses of economic outcomes and health behaviors will use generalized estimating equations to estimate the effect of the intervention at each 6-month follow-up interval and any temporal patterns in intervention effects. The secondary analysis of HIV, CT, and NG incidence between 0–12 and 12–24 months will also use generalized estimating equations.

Secondary analyses using linear models will explore the association between total savings and (1) expenditures on risky behavior including transactional sex, alcohol, and gambling and (2) investment-related expenditures (e.g., housing, schooling). As exploratory analyses of mechanisms through which the intervention may affect health and economic outcomes, we will examine the effect of the intervention on survey-based measures of future orientation, stress, and financial security. We will also undertake causal mediation analyses to further explore mechanisms of action.

### Interim analyses {21b}

N/A: No interim analyses are planned.

### Methods for additional analyses (e.g., subgroup analyses) {20b}

We will repeat the primary analysis using an interaction term between the intervention and each of 5 subgroup categories: (1) individuals who show baseline levels of more vs. less risk-seeking preferences (above/below median value), (2) different ages (above/below median age), (3) socioeconomic status based on median baseline household assets, (4) marital status (married/living as married vs. not), and (5) HIV seropositivity at baseline. For each of these five “interaction” models, we will first test the null hypothesis that the treatment effect including the interaction term explains no variation in outcome. The type I error rate will be 0.05. This analysis will help identify if the intervention unexpectedly has a negative effect on one of the subgroups and a positive effect on its complement. Next, we will describe the subgroup combinations showing the maximum benefit from the intervention using frequentist multiplicity-adjusted benefiting subgroup identification methods [36].

### Methods in analysis to handle protocol non-adherence and any statistical methods to handle missing data {20c}

Baseline characteristics of individuals with missing outcome data will be described overall and imputation will be used to impute both the predictor and outcome variables at 24 months, and models created using the imputed variables combined using standard methods. The primary analysis will use an intent-to-treat approach.

### Qualitative study analyses

We will use an interpretivist [37] qualitative analysis approach in the domain of theory-generative research. This approach involves using both “abductive” and “inductive” analysis methods to produce new empirical findings examined in light of multiple existing sociological and behavioral theories. The method is akin to constructivist grounded theory [38], in that it involves iterative rounds of coding of textual data and memo creation, but does not foreground or privilege a pre-existing heuristic or model; rather, it requires in-depth familiarity with a broad range of theories and allows for observational surprises in order to foster theory construction and generate new explanatory hypotheses. Findings will be described and displayed in tables and figures to communicate our understanding of the role of the savings intervention in participants’ spending decisions as well as in any changes in participants’ health behaviors, financial security, and planning for the future. We will also explore the intervention’s effects on the lives of female partners across multiple domains. The analysis will yield an understanding of anticipated or unanticipated positive and negative consequences of the intervention, while also elucidating observed pathways through which the intervention influences outcomes.

### Plans to give access to the full protocol, participant-level data, and statistical code {31c}

Anonymized participant datasets and statistical code will be available to other researchers upon reasonable request.

## Oversight and monitoring

### Composition of the coordinating center and trial steering committee {5d}

#### Study organization

The trial steering committee consists of members from the University of Pennsylvania, IRDO, and RTI international. They meet on a bi-weekly schedule to discuss trial implementation, progress, challenges, and study documents and to make strategic decisions. In addition to the members on the committee, the study team includes quantitative and qualitative researchers, implementation researchers, biostatisticians/methodologist, a project manager, a study coordinator, and a data manager who meet quarterly to discuss trial updates. The study coordinator meets weekly with the field staff and trained research assistants to review the study progress and challenges from the week. IRDO will be responsible for data collection and data quality and assurance. The University of Pennsylvania will be responsible for study oversight and data quality and assurance.

### Composition of the data monitoring committee, its role, and reporting structure {21a}

This study has convened a Data and Safety Monitoring Board (DSMB) to provide oversight of the trial. The role of the DSMB will be to review (a) study enrollment, (b) fidelity to the intervention, and (c) unexpected problems that might arise during the study. The DSMB is composed of experts in HIV/STI prevention, econometrics and biostatistics, medical ethics, and randomized trials. All members of the DSMB are completely independent of the investigators and have no financial, scientific, or other conflicts of interest with the trial. They will meet virtually at least once every 12 months and have any additional meetings as needed. All materials, discussions, and proceedings of the DSMB will be confidential.

### Adverse event reporting and harms {22}

In the event of a protocol deviation, serious adverse event (SAE), or unexpected incident, the investigators will promptly review the event and then notify the University of Pennsylvania Institutional Review Board (IRB) and Maseno University Ethics Review Committee within 24 h from the time of site awareness. Nonserious adverse events will be reported annually.

### Frequency and plans for auditing trial conduct {23}

Led by the study coordinator, regular trial conduct, procedural checks, and quality assurance checks will be completed by the study staff. They will verify that all documents containing protected health information are secured and align with the procedures outlined in the protocol. In addition, staff will participate in continuous training in study procedures for the duration of this study addressing any concerns or feedback from investigators, DSMB members, or participants. In addition, the study coordinator will randomly supervise staff interviews and study procedures to ensure they are being administered properly.

### Plans for communicating important protocol amendments to relevant parties (e.g., trial participants, ethical committees) {25}

IRB approvals have been obtained from the University of Pennsylvania and Maseno University Ethics Review Committee to conduct this study. Any proposed modification to the protocol, consent form, or participant-related documents will be reviewed by both Kenya- and US-based IRBs before being implemented in the field. If any amendments require amending the informed consent form, participants will be re-consented with the new informed consent form.

### Dissemination plans {31a}

Study findings will be disseminated to key stakeholders locally and internationally. Findings will also be presented at local and international meetings and conferences attended by those working on HIV prevention and treatment and behavioral economics. In addition, we will publish study results in peer-reviewed journals to ensure wider dissemination. Study participant identities will not be used in any reports or publications.

## Discussion

This project will test an innovative, theoretically motivated intervention to reduce men’s engagement in transactional sex and other risky behaviors. Leveraging innovations in mobile financial services and research on savings behavior in low-income countries, the intervention seeks to motivate high-risk, income-earning men in Kenya to reduce their spending on risky behaviors and instead save their disposable income in local bank accounts. These bank accounts will include (a) additional incentives to save in the form of lottery-based rewards linked to amounts saved, (b) opportunities to develop savings goals, and (c) periodic reminders about the incentives and goals. Through a direct economic mechanism (incentives to shift expenditures from the present to the future) and a psychological mechanism (increasing future orientation), this intervention has the potential to generate significant behavior change and improve health outcomes. By testing an intervention to promote healthy behavior among men, the findings from this study will have the potential to address a missing element of HIV/STI prevention efforts in SSA. By seeking to reduce men’s engagement in high-risk behaviors like transactional sex and alcohol use, the study addresses “upstream” factors that can affect men’s and women’s health outcomes.

Due to the highly gendered labor markets in SSA, men are at an economic advantage relative to women, with men being more likely to engage in skilled labor and to earn regular income. While this contributes to a power imbalance and transactional sex, it also reveals why a savings intervention may be especially appropriate for men in this context. Savings interventions have the potential to incentivize men to reduce their spending on transactional sex and alcohol while concurrently saving it for the future, in a similar way that cash transfers may help women reduce their reliance on transactional sex. This study expands on a pilot trial we conducted in Kenya among men who were engaged in transactional sex, which found that men were interested in obtaining savings accounts and used them to increase their savings [7]. Given these preliminary data, larger-scale evaluation of a savings intervention will reveal whether this approach is effective in displacing entrenched social norms, specifically in a population with limited access to financial services and high levels of engagement in HIV risk behaviors.

There are a number of benefits associated with this study and minimal risks. Participants in both study groups will benefit from access to free HIV and other STI testing. Participants who receive a positive result will benefit from receiving free treatment of curable STIs and referral to care and treatment for HIV. Participants in the intervention group will also benefit from the opportunity to receive a bank account and obtain lottery-based interest if they save money. Those who save more money can experience an increase in financial security and a decrease in financial stress. There are a few minimal risks involved in this study. Participants may experience some discomfort or pain during sample collection. Participants may also feel uncomfortable providing information about their sexual history and spending habits with the study team. In addition, it is possible that participant’s family or friends may find out about their involvement in the study. These minimal risks faced by participants in this study are likely to be far outweighed by the benefits to participants and the community, as well as the importance of the knowledge to be gained from the study.

The study has several strengths that are worth noting. These include a rigorous experimental design to generate robust, unbiased results of the intervention’s effect on health and economic outcomes, intent-to-treat analyses, objective outcome measures, adequate statistical power, and exploration of mechanisms using quantitative and qualitative methods. To our knowledge, this will be the first study to assess whether savings interventions affect men’s health behavior in a setting with high HIV prevalence. The study also has several limitations. First, some outcomes are self-reported (i.e., overall savings, sexual behavior, alcohol use). Second, if the savings intervention were to have a weak effect on savings behavior, it will undermine our ability to observe changes in risky behaviors. It is also possible that if some participants receive large rewards from saving money, their spending on risky behaviors may increase rather than decrease. Some men may also divert spending on food and non-food items towards savings accounts. We expect this to be unlikely based on our pilot study data, but we will empirically examine this possibility and consult our DSMB to develop protective measures (e.g., reducing larger prize amounts) if adverse effects are detected.

In summary, this study will make three major contributions. First, it will evaluate whether prize-linked savings interventions reduce risky health behaviors and HIV/STI incidence among men. Second, by assessing impacts on savings behavior, the project will simultaneously address a policy priority of expanding financial services and combatting poverty. Third, with quantitative and qualitative methods, it will advance our understanding of mechanisms by which savings interventions affect health behavior.

At study completion, we expect to generate the first estimates of the effect of savings-led interventions on HIV risk behaviors among men in SSA. The study has the potential to break new ground on ways to reduce engagement in transactional sex and heavy alcohol use among men. Overall, the study promises high scientific and public health impact by testing an innovative, multi-sectoral, HIV status-neutral approach to increasing savings and reducing risky health behaviors among men.

## Trial status

Enrollment of study participants began on July 18, 2022, and is expected to be completed by March 2023. Follow-up visits are expected to begin in January 2023 and end in March 2025. The current protocol is version 4.0 as of September 15, 2022.

## Data Availability

Data collected from participants will be available to researchers who provide a methodologically sound proposal. Requests should be directed to the principal investigator (hthirumu@pennmedicine.upenn.edu).

## Abbreviations

ART: Antiretroviral therapy
CT: *Chlamydia trachomatis*
DSMB: Data and Safety Monitoring Board
HIV: Human immunodeficiency virus
HSV-2: Herpes simplex virus type 2
IDI: In-depth interview
IRB: Institutional review board
IRDO: Impact Research and Development Organization
NASCOP: National AIDS and STI Control Program
NG: *Neisseria gonorrhoeae*
PrEP: Pre-exposure prophylaxis
REDCap: Research Electronic Data Capture
SAE: Serious adverse event
SSA: Sub-Saharan Africa
STI: Sexually transmitted infection
